# Polymorphisms in Predicted miRNA Binding Sites and Osteoporosis

**DOI:** 10.1002/jbmr.186

**Published:** 2010-07-16

**Authors:** Shu-Feng Lei, Christopher J Papasian, Hong-Wen Deng

**Affiliations:** 1 Laboratory of Molecular and Statistical Genetics and the Key Laboratory of Protein Chemistry and Developmental Biology of the Ministry of Education, College of Life Sciences, Hunan Normal UniversityChangsha, Hunan, People's Republic of China; 2 Department of Basic Medical Science, School of Medicine, University of Missouri–Kansas CityKansas City, MO, USA; 3 Human Genetics/Genomics Program, Department of Orthopedic Surgery, School of Medicine, University of Missouri–Kansas CityKansas City, MO, USA; 4 Center of Systematic Biomedical Research, University of Shanghai for Science and TechnologyShanghai, People's Republic of China; 5 College of Life Sciences and Bioengineering, Beijing Jiaotong UniversityBeijing, People's Republic of China

**Keywords:** MICRORNA, OSTEOPOROSIS, ASSOCIATION, POLYMORPHISM

## Abstract

MicroRNAs (miRNAs) regulate posttranscriptional gene expression usually by binding to 3'-untranslated regions (3'-UTRs) of target message RNAs (mRNAs). Hence genetic polymorphisms on 3'-UTRs of mRNAs may alter binding affinity between miRNAs target 3'-UTRs, thereby altering translational regulation of target mRNAs and/or degradation of mRNAs, leading to differential protein expression of target genes. Based on a database that catalogues predicted polymorphisms in miRNA target sites (poly-miRTSs), we selected 568 polymorphisms within 3'-UTRs of target mRNAs and performed association analyses between these selected poly-miRTSs and osteoporosis in 997 white subjects who were genotyped by Affymetrix Human Mapping 500K arrays. Initial discovery (in the 997 subjects) and replication (in 1728 white subjects) association analyses identified three poly-miRTSs (rs6854081, rs1048201, and rs7683093) in the fibroblast growth factor 2 (*FGF2*) gene that were significantly associated with femoral neck bone mineral density (BMD). These three poly-miRTSs serve as potential binding sites for 9 miRNAs (eg, miR-146a and miR-146b). Further gene expression analyses demonstrated that the *FGF2* gene was differentially expressed between subjects with high versus low BMD in three independent sample sets. Our initial and replicate association studies and subsequent gene expression analyses support the conclusion that these three polymorphisms of the *FGF2* gene may contribute to susceptibility to osteoporosis, most likely through their effects on altered binding affinity for specific miRNAs. © 2011 American Society for Bone and Mineral Research.

## Introduction

Osteoporosis is a problem of excessive skeletal fragility leading to large numbers of low-trauma fractures among the elderly. Osteoporosis and its major risk factors, such as low bone mineral density (BMD), are under strong genetic determination.(1–3) The major goal in genetic studies of osteoporosis is to identify causal genetic variations underlying osteoporosis. Genetic mutations that alter protein sequences and polymorphisms that regulate gene expression (ie, regulatory polymorphisms) are two naturally occurring genetic variations that are identified and characterized often when studying the genetics of complex diseases/traits.(4,5)

MicroRNAs (miRNAs) are endogenous noncoding RNAs containing 21 to 23 nucleotides that regulate posttranscriptional gene expression, usually by binding to 3'-untranslated regions (3'-UTRs) of target message RNAs (mRNAs).(6,7) Therefore, polymorphisms in 3'-UTRs of mRNAs potentially could alter the affinity of miRNAs for their target mRNAs. This altered affinity could affect the efficiency with which miRNAs regulate protein expression by altering their capacity to repress mRNA translation and/or promote mRNA decay. Regulatory polymorphisms in miRNA target sites (poly-miRTSs) are being widely and actively studied for their contribution to genetic variations in human diseases ranging from Parkinson disease to cancer.(8–12) Several significant poly-miRTSs associated with cancers have been identified(8–11); for example, a significant association was found between the risk of non–small cell lung cancer and a polymorphism in the let-7 miRNA complementary site in the 3'-UTR of the *KRAS* gene.(11) To date, however, genetic studies have not been attempted to identify poly-miRTSs that may be associated with osteoporosis risk.

Recently, a poly-miRTS database identified and catalogued naturally occurring genome-wide DNA variations in putative miRNA target sites.(13) These predicted poly-miRTSs may affect the regulation of target mRNA by miRNA and contribute to phenotypic differences between individuals that alter the risk for complex diseases such as osteoporosis. We selected a total of 568 poly-miRTSs that were genotyped recently using Affymetrix Human Mapping 500K SNP arrays (Affymetrix, Santa Clara, CA, USA) in a discovery sample of 997 unrelated white people and performed association analyses to investigate the potential role of these identified poly-miRTSs on osteoporosis. The initial discovery and replication (in an independent sample 1,728 white subjects) association studies (DNA level), along with subsequent gene expression analyses (RNA level), identified three single-nucleotide polymorphisms (SNPs) in the 3'-UTR of the fibroblast growth factor 2 (*FGF2*) gene as putative binding sites for miRNAs that may contribute to the risk of osteoporosis.

## Materials and Methods

### Samples

The study was approved by the necessary institutional review board or research administration of the involved institutions. Signed informed-consent documents were obtained from all study participants before entering the study. Detailed characteristics of studied subjects are summarized in Tables 1 and 4.

**Table 1:** Basic Characteristics of Association Study Subjects

Trait	Initial study (*n* = 997)	Replicate study (*n* = 1728)
Age (years)	50.3 (18.3)	51.6 (12.9)
Height (cm)	170.8 (9.7)	163.3 (6.3)
Weight (kg)	80.2 (17.8)	71.4 (16.0)
Femoral neck BMD (g/cm^2^)	0.814 (0.145)	0.793 (0.133)

*Note:* Data are shown as mean (SD).

### Association study samples

#### Initial discovery association sample

A total of 997 unrelated subjects (aged 50.3 ± 18.3 years) were identified from our established and ever-expanding database containing more than 7000 subjects for the initial discovery study. All identified subjects were US white people of European origin living in the Midwestern United States in Omaha, Nebraska. This sample was recruited with the aim of identifying genes that influence the risk of various complex diseases (e.g., osteoporosis, obesity, and sarcopenia) and several important complex traits by approaches such as genome-wide association analyses.(14–19) Strict exclusion criteria were adopted to minimize any known potential confounding effects on bone phenotype variations. Briefly, patients with chronic diseases/conditions that potentially may affect bone mass were excluded. These diseases/conditions included chronic disorders involving vital organs (eg, heart, lung, liver, kidney, and brain), serious metabolic diseases (eg, diabetes, hypo- or hyperparathyroidism, and hyperthyroidism), other skeletal diseases (eg, Paget disease, osteogenesis imperfecta, and rheumatoid arthritis), chronic use of drugs affecting bone metabolism (eg, corticosteroid therapy and anticonvulsant drugs), and malnutrition conditions (eg, chronic diarrhea and chronic ulcerative colitis). Femoral neck (FN) bone mineral density (BMD) was measured using Hologic densitometers (Hologic, Inc., Bedford, MA, USA).

#### Replication association sample

This sample contained 1,728 unrelated white women (aged 51.6 ± 12.9 years). All selected subjects were US white women of European origin. All identified subjects were US white women of European origin living in the midwestern United States in Omaha (Nebraska) and Kansas City (Missouri). This sample was recruited with the aim to identify genes that influence the risk of various complex diseases (eg, osteoporosis, obesity, and sarcopenia). This sample was independent from the sample used in the initial discovery association study.

### Gene expression samples

Expression data for the *FGF2* gene were obtained during studies with three independent gene expression samples (Caucasians 1, 2, and 3 described below) with high versus low hip BMD. These samples were recruited with an original purpose of systemically searching for differentially expressed genes underlying BMD variation (published data from the first sample(20) and unpublished data from the other two samples). The first sample (Caucasian 1) consisted of 9 premenopausal white women. The second sample (Caucasian 2) contained 40 unrelated white women with high hip BMD and 40 matched white women with low hip BMD. The third sample (Caucasian 3) consisted of 79 unrelated white women, including 39 with high and 40 with low hip BMD. Hip BMD value is expressed as *Z*-score that is defined as the number of standard deviations of a BMD measurement above (ie, a positive *Z*-score) or below (ie, a negative *Z*-score) the age-, gender-, and ethnicity-matched population mean BMD. In order to minimize any known potential confounding effects on the variation in bone phenotype, these three samples were recruited by adopting strict exclusion criteria that were similar to those used to recruit the sample for the initial discovery association study. Circulating monocytes and B cells were used in these gene expression studies because of their important role in osteoclastogenesis. Circulating monocytes serve as progenitors of osteoclasts(20–23) and also produce a wide variety of factors involved in bone metabolism, such as interleukin 1 (IL-1), tumor necrosis factor α (TNF-α), IL-6, platelet-derived growth factor, transforming growth factor β (TGF-β), and 1,25-dihydroxyvitamin D_3_ [1,25(OH)_2_D_3_].(24,25) B cells may participate in osteoclastogenesis through expression of osteoclast-related factors, such as RANKL, TGF-β, and osteoprotegerin (OPG).(26)

### Experimental procedures

#### DNA extraction and genotyping

Genomic DNA was extracted from whole human blood using a commercial isolation kit (Gentra Systems, Minneapolis, MN, USA) according to the kit protocol. Subjects used in the initial discovery association study were genotyped using Affymetrix Human Mapping 500K arrays, which examined approximately 500,000 SNPs. Genotyping with the Affymetrix Mapping 250K Nsp and Affymetrix Mapping 250K Sty arrays was performed at the Vanderbilt Microarray Shared Resource at Vanderbilt University Medical Center (Nashville, TN, USA) using the standard protocol recommended by the manufacturer. Fluorescence intensities were quantified using an Affymetrix Array Scanner 30007G. Dynamic model (DM)(27) calls were used for quality control of the genotyping experiment. Genotyping was performed initially for 1000 US whites, and unsatisfactory arrays were subject to regenotyping. Eventually, 997 subjects who had at least one array (Nsp or Sty) reaching a 93% call rate were retained. Subjects used in the replication association study were genotyped using the Genome-Wide Human SNP Array 6.0 following the standardized procedures recommended by the manufacturer.

#### Isolation of monocytes and B cells

A monocyte-negative isolation kit (Dynal Biotech, Inc., Lake Success, NY, USA) was used to isolate circulating monocytes from 50 mL of whole blood following the procedures recommended by the manufacturer. B-cell isolation from 70 mL of whole blood was performed using a positive isolation method with Dynabeads CD19 (Pan B) and DETACHaBEAD CD19 (Dynal Biotech) following the manufacturer's protocols.

### Total RNA extraction and microarray assay

Total RNA was extracted from monocytes and B cells using a Qiagen kit (Qiagen, Inc., Valencia, CA, USA) following the procedures recommended by the manufacturer. Experimental procedures for gene expression microarray assays were performed according to the manufacturer's protocol (Affymetrix). Briefly, RNA was converted to double-stranded cDNA. In vitro transcription was performed to produce biotin-labeled cRNA (BioArray HighYield RNA Transcription Labeling Kit, Enzo Diagnostics, New York, NY, USA). Biotinylated cRNA was cleaned, fragmented, and hybridized (Affymetrix Genechip Hybridization Oven 640) to U133 A Gene Chips. Then microarrays were washed (Affymetrix Fluidics Station 450), stained with phycoerythrin-streptavidin, and scanned using an Affymetrix scanner (Gene Array Scanner 3000).

### Statistical analysis

GeneChip Operating Software (GCOS) was used to control GeneChip fluidics stations and scanners (Affymetrix, Santa Clara, CA, USA), acquire data, manage sample and experimental information, and generate the raw array data in CEL files. We used the Robust Multiarray Average (RMA) algorithm(28) to transform the probe-level raw data into gene expression data. RMA can provide the most reproducible results and shows the highest correlation coefficients with RT-PCR data among currently available algorithms.(29) Based on expression data generated with the RMA algorithm, Student's *t* test was used to compare expression signals in subject groups with low versus high BMD to identify differentially expressed genes.

### Poly-miRTS identification and selection

The method of identifying and annotating poly-miRTS was detailed by Bao and colleagues.(13) Briefly, we first extracted SNPs that are located in 3'-UTRs of all known human genes from dbSNP build 126.(30) Genomic locations for these SNPs then were mapped onto mRNAs, and the criteria of TargetScanS(31) were used to predict miRNA target sites. About 22,000 human predicted poly-miRTSs were collected from the poly-miRTS database. Of these, 860 poly-miRTSs were genotyped using Affymetrix 500K SNP arrays in the 997 unrelated white subjects. Among the 860 genotyped poly-miRTSs, 292 were excluded because of the following quality-control criteria: Allele frequency deviated extremely from Hardy-Weinberg equilibrium (*p* < .001), or minor allele frequency (MAF) was less than 1%. Eventually, 568 poly-miRTSs were selected for subsequent association analyses.

### Association analysis

Raw values were adjusted by four significant covariates (ie, age, gender, height, and weight). PLINK (http://pngu.mgh.harvard.edu/∼purcell/plink/) performed linear regression analyses to evaluate associations between the 568 selected poly-miRTSs and femoral neck BMD. *p* values and regression coefficients (β value) produced from linear regression analyses are two important parameters for assessing associations.

### SNP imputation and association testing

Since some of DNA samples for the original discovery study were not available, it was not easy to retrospectively genotype, in the initial discovery sample, the two interesting poly-miRTSs (rs1048201 and rs7683093) that were genotyped in the replicate study using Genome-Wide Human SNP Array 6.0 but were not genotyped directly in the initial discovery sample using Affymetrix Human Mapping 500K array. However, SNP imputation is a reasonable alternative method for inferring the ungenotyped SNPs. In order to perform association testing for these two interesting poly-miRTSs, imputation was performed by PLINK functions (http://pngu.mgh.harvard.edu/∼purcell/plink/pimputation.shtml) based on a reference panel from HapMap. The process of SNP imputation and association testing included the following: (1) finding flanking markers and haplotypes (proxies) that are in strong linkage disequilibrium with the reference SNPs and (2) testing these proxies for associations with diseases within a haplotype-based framework.

## Results

### Association of poly-miRTS and osteoporosis

The basic characteristics of association study subjects are listed in Table 1. We identified 7 poly-miRTSs in our discovery sample that manifested association signals with femoral neck BMD at the significance level of *p* < .01; these 7 poly-miRTSs are potential binding sites for 12 distinct miRNAs (eg, miR-146a and miR-146b) (Table 2). The strongest association signal for femoral neck BMD was detected at poly-miRTS rs1712 in the F-box protein 5 (*FBXO5*) gene (*p* = 2.54E-03); this poly-miRTS is located at a predicted binding site for miR-549. In the replication study, with an independent white sample, associations with femoral neck BMD were replicated only for two of these SNPs (rs1712 and rs6854081). However, the association direction for the regression coefficient (β value) of rs1712 differed between the initial discovery and replicate studies (Table 2). For rs6854081, a poly-miRTS in the 3'-UTR of the *FGF2* gene, the association direction for the regression coefficient was the same in both the initial and replicate studies, so our subsequent analyses focused on rs6854081 in the *FGF2* gene.

**Table 2:** Associations of Poly-miRTSs With Femoral Neck BMD in the Initial and Replicate Studies

					Initial study^d^		Replicate study^d^			
								
SNP ID	Allele^b^	Chromosome	Physical position	MAF^a^	*p*	β^e^	*p*^c^	β^e^	Gene symbol	Potentially associated miRNA
rs6854081	*G/T*	4q28.1	124036157	0.131	8.37E-03	–0.020	5.79E-02*	–0.010	*FGF2*	miR-146a, miR-146b
rs1712	*T/C*	6q25.2	153333558	0.159	2.54E-03	–0.021	3.50E-02	0.010	*FBXO5*	miR-549
rs10518716	*C/G*	15q22.33	65281877	0.224	5.05E-03	0.017	4.38E-01	–0.003	*FLJ11506*	miR-380-5p, miR-563
rs17054320	*T/A*	6q25.1	151204546	0.039	5.07E-03	0.037	6.72E-01	–0.004	*PLEKHG1*	miR-212, miR-132 miR-505, miR-421
rs10793442	*T/G*	10q11.21	43371825	0.129	5.97E-03	–0.021	5.59E-01	0.003	*ZNF239*	miR-361
rs10098470	*T/C*	8q21.13	81111833	0.029	7.58E-03	0.041	6.62E-01	0.005	*TPD52*	miR-582
rs2745426	*A/G*	6q23.2	133045037	0.275	9.88E-03	0.015	8.09E-01	–0.001	*VNN1*	miR-302b

a MAF = minor allele frequency in the initial study sample.

b The first allele represents the minor allele of each SNP.

c Asterisk (*) = the direction of regression coefficient in the replication study was the same as that in the initial association, so the *p* value for significant replication association is *p* < .1.

d The initial discovery sample was genotyped using Affymetrix Human Mapping 500K Arrays, and the replication study sample was genotyped using Genome-Wide Human SNP Array 6.0.

e For the additive effects of SNPs, the direction of the regression coefficient represents the effect of each extra minor allele; that is, a positive regression coefficient means that the minor allele increases the mean BMD phenotype.

**Fig. 1:**
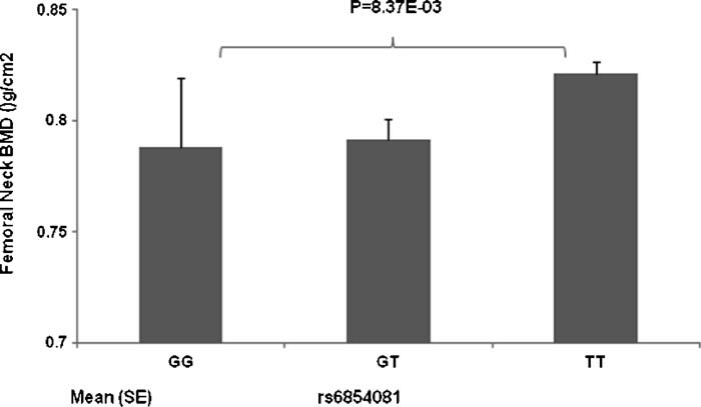
Distribution of raw femoral neck BMD stratified by rs6854081 genotypes in the discovery study.

As shown in Fig. 1, subjects with a homozygous *TT* genotype for rs6854081 had a higher raw femoral neck BMD than individuals with a homozygous *GG* genotype. Fisher's combined *p* value(32) from the two independent studies is more significant (*p* = 4.18E-03) than the *p* values for each independent study (Table 2). By searching the poly-miRTS database, we found two additional poly-miRTSs (rs1048201 and rs7683093) in the *FGF2* gene that were genotyped in the replicate study using Genome-Wide Human SNP 6.0 Arrays but were not genotyped directly in the initial discovery sample using Affymetrix Human Mapping 500K arrays. Therefore, we performed further association analyses for these two SNPs in the replication sample and found that both rs1048201 and rs7683093 were significantly associated with femoral neck BMD in the replicate sample. We also performed SNP imputation and association testing for these two additional poly-miRTSs in the initial sample and found that rs1048201, but not rs7683093, was significantly associated with femoral neck BMD in the discovery sample. The three significant SNPs (rs6854081, rs1048201, and rs7683093) reside within 9 predicted miRNA target sites (Table 3).

**Table 3:** Poly-miRTSs in the *FGF2* Gene and Associated miRNAs

					Initial study			Replicate study				
								
SNP ID	Allele^a^	Physical position	SNP position in RNA	Perfect match allele	MAF^b^	β	*p*^c^	MAF^b^	β	*p*	Allele miRSeed	Potentially associated miRNA
rs6854081	*G/T*	124036156	4088	*T*	0.131	−0.020	8.37E-03	0.138	−0.010	5.79E-02	*GTTCTC*	miR-146a, miR-146b
rs1048201	*T/C*	124033757	1691	*T*	0.250	1.640	4.51E-02	0.178	0.009	4.68E-02	*TGCTGA*	miR-545
rs7683093	*C/G*	124037534	5466	*G*	0.140	0.760	3.76 E-01	0.136	−0.010	4.21E-02	*TGCAAT*	miR-25, miR-32, miR-92, miR-363, miR-367, miR-92b

a The first allele represents the minor allele of each SNP.

b MAF = minor allele frequency in the study sample.

c The last two poly-miRTSs (rs1048201 and rs7683093) were not genotyped directly in the initial discovery sample using Affymetrix Human Mapping 500K Arrays but were genotyped directly in the replicate study using Genome-Wide Human SNP Array 6.0. SNP imputation and association testing for these two SNPs were performed by PLINK functions in the initial sample.

### Gene expression of the *FGF2* gene

To further investigate the relevance of the *FGF2* gene to osteoporosis and to provide complementary evidence supporting the associations detected earlier, we performed differential expression analyses for the *FGF2* gene in three distinct gene expression studies (Table 4). These three studies compared *FGF2* gene expression in subjects with high versus low BMD using two distinct cell types related to bone metabolism (ie, B-lymphocytes and monocytes). Two expression probes (204421_s_at and 204422_s_at) were specifically designed to detect expression of the *FGF2* gene in the Affymetrix Human Genome U133 A Array. There was significant or nearly significant differential expression in low versus high hip BMD for at least one of these probes in each sample studied (Table 4). Moreover, in all three samples, these probes consistently indicated decreased expression of the *FGF2* gene in the high versus low BMD groups.

**Table 4:** Differential Expression of Probes in the *FGF2* Gene in Three Female Samples

		High BMD group				Low BMD group					
					
Sample	Cell	*N*	Age	Hip BMD^a^	Expression level	*N*	Age	Hip BMD^a^	Expression level	Probe	*p* Value
Caucasian 1	Monocyte	5	49.4 (3.2)	1.77 (0.99)	3.96	4	50.5 (2.9)	–0.79 (0.13)	4.10	204422_s_at	4.89E-02
Caucasian 2	Monocyte	40	49.4 (8.1)	1.45 (0.67)	5.71	40	50.0 (7.9)	–1.05 (0.44)	5.77	204421_s_at	7.06E-03
Caucasian 3	B cell	39	49.6 (8.0)	1.42 (0.67)	4.49	40	50.2 (7.9)	–1.05 (0.44)	4.51	204422_s_at	5.18E-02

*Note:* All subjects were profiled by Affymetrix Human Genome U133 A arrays. The RMA algorithm was used to transform the probe-level intensity data into gene expression data. Student's *t* test was performed to compare the expression data generated with the RMA algorithm in two groups of each sample. All probes consistently indicated decreased expression in high versus low BMD groups in all three samples.

a *Z*-score of hip BMD.

## Discussion

This study reports the important role of polymorphisms in 3'-UTRs of mRNAs (miRNA-binding sites) in determining variations in BMD. We have identified three significant poly-miRTSs that are associated with BMD in the 3'-UTR of the *FGF2* gene. These findings are supported by three independent gene expression analyses that consistently demonstrated depressed expression of the *FGF2* gene in subjects with high BMD compared with subjects with low BMD.

Fibroblast growth factor is well known for its functional effects on bone biology. It plays a key role in the development of the bone matrix and regulation of bone remodeling and has both direct and indirect effects on osteoclast formation and bone resorption.(25,33,34) Specifically, fibroblast growth factor is thought to stimulate osteoclast recruitment, development, and bone pit resorption.(25) Despite the functional importance of FGF2 on bone, to the best of our knowledge, no previous association studies have demonstrated a relationship between polymorphisms of the *FGF2* gene and bone phenotypes (eg, BMD). In this study, we report, for the first time, that three polymorphisms of the *FGF2* gene are consistently associated with BMD. The data from association analyses at the DNA level and gene expression analyses at the RNA level are consistent in supporting the concept that polymorphisms detected in the *FGF2* gene regulate BMD variation, possibly by altering the affinity of mRNA products for specific miRNAs.

Based on the results from our association and expression analyses, we would hypothesize the following potential mechanism by which poly-miRTSs regulate BMD through miRNA. Using rs6854081 as an example, miR-146a and miR-146b would bind optimally to *FGF2* mRNA transcripts containing allele *T* at rs6854081. This optimal binding would be expected to negatively regulate protein expression by promoting mRNA degradation and/or repressing mRNA translation. Conversely, binding of these miRNAs to mRNA transcripts with allele *G* would not be as strong and therefore would allow higher levels of protein expression. Higher levels of FGF protein would be expected to stimulate osteoclastogenesis through osteoclast formation and late differentiation. This would enhance bone resorption, leading to lower BMD, which is consistent with findings of our gene expression studies; that is, high expression of the *FGF2* gene results in low BMD. Consequently, we can infer that individuals with the *TT* genotype should have higher BMD than subjects with the *GG* genotype, which is consistent with the actual observation presented in Fig. 1. Extensive functional studies will be required to confirm this proposed hypothetical mechanism by which poly-miRTSs regulate BMD.

In the current initial discovery sample, we used multiple approaches, described previously,(35) to detect population stratification that may lead to spurious association results. The software Structure 2.2 (http://pritch.bsd.uchicago.edu/software.html) analyzed up to 10,000 unlinked markers and found that the vast majority (98%) of subjects were tightly clustered together. The *inflation factor* λ, calculated by Genomic Control,(36) was 1.007, indicating that potential population stratification in this homogeneous US white population is very minimal. This combined evidence suggests that the confounding effects from population structure, if present, contribute very little to our initial association results.

Among the identified poly-miRTSs collected in the database,(13) only 568 poly-miRTSs were assessed for potential associations with osteoporosis in this study. Most of the poly-miRTSs (∼22,000) identified in the database were not covered by either the Affymetrix Human Mapping 500K Array or the Genome-Wide Human SNP Array 6.0. Consequently, there is an obvious need for better coverage of SNPs in 3'-UTRs in future commercial SNP arrays. As the technology advances, further studies will be needed to evaluate how osteoporosis is affected by the SNPs that were included in the poly-miRTS database(13) but were not analyzed in this study. These future studies should provide additional insights into the importance of polymorphisms in 3'-UTRs of mRNAs on regulation of gene expression as it relates to bone biology.

Examining the role of miRNAs in regulating bone represents a new frontier in bone biology that we are just beginning to explore. This study is, to the best of our knowledge, the first effort to examine the role of miRNA target-site polymorphisms in regulating bone, and these initial findings may be of significant interest to the bone community. The results presented represent an initial logical step toward understanding whether miRNA target-site polymorphisms play a role in bone remodeling. In the future, extensive efforts will be required to fully disclose the underlying biologic mechanisms for the associations between the predicted poly-miRTSs and bone phenotypes. These efforts may include the following: (1) Correlations between genotypes of poly-miRTSs and expression of the *FGF2* gene at mRNA and protein levels should be established in a large sample, with the purpose of gaining an understanding of the potentially different effects that poly-miRTSs may have on regulating expression of the *FGF2* in vivo. (2) Tests should be conducted to determine if specific miRNAs actually regulate expression of the *FGF2* gene by binding to predicted target sequences. Luciferase reporter assays would be a very good method for achieving this goal (detailed in ref. 12). By constructing allele-specific *Renilla* luciferase plasmids containing one of the alleles of the poly-miRTS, this method also can test the allele-specific effects of miRNA on regulating expression of *FGF2*. (3) Polymorphisms in 3'-UTRs of mRNAs potentially could alter the affinity of miRNAs for target mRNAs, and this altered affinity could affect the efficiency with which miRNAs regulate protein expression by promoting mRNA decay and/or by repressing mRNA translation. Therefore, it is necessary to determine whether poly-miRTSs have different effects on regulating mRNA degradation of the *FGF2* gene by measuring mRNA half-life via quantitative RT-PCR analyses. Should all the preceding proposed analyses be confirmed, a conclusion about how poly-miRTSs regulate variation of bone phenotypes will be made with high confidence and resolution.

In conclusion, we have detected several putative osteoporosis-associated poly-miRTSs in this study. Although we have not specifically determined the biologic mechanisms underlying these associations, our results should be of interest to the bone community because the detected polymorphisms might be worthy candidates for future functional studies. It is anticipated that future large-scale genome-wide scans for poly-miRTSs and follow-up functional experiments will identify additional poly-miRTSs involved in the pathogenesis of osteoporosis and the specific mechanisms by which they exert their biologic influence.
